# Ethological Evaluation of the Effects of Social Defeat Stress in Mice: Beyond the Social Interaction Ratio

**DOI:** 10.3389/fnbeh.2015.00364

**Published:** 2016-02-03

**Authors:** Aron M. Henriques-Alves, Claudio M. Queiroz

**Affiliations:** Brain Institute, Federal University of Rio Grande do NorteNatal, Brazil

**Keywords:** resident–intruder paradigm, social defeat stress, stretch-attend posture, flight, defensive behaviors, sucrose preference test, phenotyping

## Abstract

In rodents, repeated exposure to unavoidable aggression followed by sustained sensory treat can lead to prolonged social aversion. The chronic social defeat stress model explores that phenomenon and it has been used as an animal model for human depression. However, some authors have questioned whether confounding effects may arise as the model also boosts anxiety-related behaviors. Despite its wide acceptance, most studies extract limited information from the behavior of the defeated animal. Often, the normalized occupancy around the social stimulus, the interaction zone, is taken as an index of depression. We hypothesized that this parameter is insufficient to fully characterize the behavioral consequences of this form of stress. Using an ethological approach, we showed that repeated social defeat delayed the expression of social investigation in long (10 min) sessions of social interaction. Also, the incidence of defensive behaviors, including stretched-attend posture and high speed retreats, was significantly higher in defeated mice in comparison to controls. Interestingly, a subpopulation of defeated mice showed recurrent and non-habituating stretched-attend posture and persistent flights during the entire session. Two indexes were created based on defensive behaviors to show that only recurrent flights correlates with sucrose intake. Together, the present study corroborates the idea that this model of social stress can precipitate a myriad of behaviors not readily disentangled. We propose that long sessions (>150 s) and detailed ethological evaluation during social interaction tests are necessary to provide enough information to correctly classify defeated animals in terms of resilience and susceptibility to social defeat stress.

## Introduction

Social stress is considered a major risk factor for the onset and development of neuropsychiatric disorders (Charney and Manji, 2004; Sayed et al., 2015). It has been suggested that genetics and developmental factors can contribute to determine whether the individual will develop depression, anxiety, bipolar disorder, or schizophrenia (or comorbidity between them) after stressful events (Turner et al., 1995; Zelena et al., 1999; Connor-Smith and Compas, 2002; Southwick et al., 2005; Vidal et al., 2007). Despite being often treated as single and separate clinical entities, such disorders share some important pathological behaviors, including reduced social interaction (social phobia, aversion, or withdrawal) and anhedonia (Gorman, 1996; Kessler et al., 1999; Pizzagalli, 2014). In fact, comorbidity rates are found to be high among stress-related psychopathologies, for example, in mood and anxiety disorders (Gorman, 1996; Mineka et al., 1998; Waugh et al., 2012). Interestingly, not all stress-suffering individuals develop one or another pathology and, in fact, most of them are resilient to a certain degree of stress (Southwick et al., 2005; Southwick and Charney, 2012), suggesting that genetic background and family history can help to cope with social stress (Feder et al., 2009).

Most of our understanding of the mechanisms involved in the vulnerability to social stress has come from studies performed in animals (Nestler and Hyman, 2010). Animal models of social stress involve subjecting rodents to brief episodes of social subordination and aggression by a larger and more aggressive conspecific (Miczek, 1979; Kudryavtseva et al., 1991; Koolhaas et al., 1997). After repeated exposure to confrontations, rats and mice show a wide range of depression-like symptoms, including anhedonia and social avoidance (Kudryavtseva et al., 1991; Krishnan et al., 2007). Like humans, anxiety-like symptoms are also observed in a subgroup of individuals (Krishnan et al., 2007). Importantly, these stress-induced behaviors develop differently in subjects, which makes the social defeat model useful to study resilience and susceptibility to stress (Krishnan et al., 2007; Nestler and Hyman, 2010).

It has been argued that understanding human psychiatric disorders depends on better animal models (Nestler and Hyman, 2010), not only through the development of new experimental approaches but mainly by improving the characterization of the pathological behavior (Fonio et al., 2012b; Toth and Neumann, 2013). Traditionally, social behaviors are commonly evaluated by using social interaction tests (File and Seth, 2003; Golden et al., 2011) which are intended to measure the amount of time the experimental animal has spent interacting with an unfamiliar conspecific (Kudryavtseva et al., 1991; Berton et al., 2006; Krishnan et al., 2007; Venzala et al., 2012). However, an indirect index of social interaction is used instead. In most studies, it is calculated as a ratio between the amount of time spent in the close vicinity of the social stimulus (i.e., the interaction zone) and the time spent in the same area in the absence of any social cue during short-duration (~150 s) sessions (Krishnan et al., 2007; Yin et al., 2015). Also, recent work has suggested that the identification of behavioral phenotypes in relatively short duration tests can have confounding effects due to acute experimental procedures and/or novelty-induced anxiety (Fonio et al., 2012a; Hager et al., 2014). This is especially true when the emotional state of the animals is under scrutiny. Another important issues include an almost completely absence of information regarding defeat variables (i.e., latency to attack and number of attacks) which could add to the inter-individual variability in the behavioral outcome (Chaouloff, 2013) and lack of specificity of some antidepressants for depressive- and anxiety-related behaviors induce by the defeat (Berton et al., 1999; Venzala et al., 2012). For these reasons, more sophisticated behavioral approaches are necessary to evaluate how changes in animal behavior triggered by social defeat relates to human neuropsychiatric disorders (Peters et al., 2015).

In this context, many research groups have been using ethologically-oriented measures to enhance biological significance of behavioral tests (Blanchard and Blanchard, 1988; Kshama et al., 1990; Rodgers and Johnson, 1995; Sorregotti et al., 2013; Hager et al., 2014; Peters et al., 2015). In this respect, quantification of defensive behaviors, such as stretched-attend postures and flights, are quite informative in social interaction tests. Stretched-attend posture occurs during risk assessment and is often observed when the animal is not sure about the presence and/or location of the threat source (Grant and Mackintosh, 1963; Blanchard and Blanchard, 1988). In mice, stretched-attend posture is characterized by the elongation of the forepart of the animals' body toward unknown stimuli while the animal keeps a relative safe distance from the possible threat (Augustsson and Meyerson, 2004; Hager et al., 2014)—a highly adaptive behavior—relevant for the correct choice of defensive possibilities such as fleeing (flight), freezing, or attacking defensively (Eilam, 2005; Stankowich and Blumstein, 2005; Blanchard et al., 2011). However, behavioral defensive states are, metabolically speaking, costly (McEwen et al., 2015) and therefore, correct identification of possible threats is crucial to allow the return to non-defensive behaviors (Blanchard and Blanchard, 1988; Blanchard et al., 2011). Our working hypothesis states that depression- and anxiety-related behaviors in mice reflect the difficulty that the experimental animal has in evaluating reward and threat respectively, and ethologically-oriented measures can be used to disentangled these profiles (Peters et al., 2015). To test that, we have performed 10 min long sessions of the social interaction tests to characterize the time-course of social investigation and risk assessment behavior in repeated (5 days) socially defeated mice. We have introduced novel indexes based on defensive behaviors to segregate and differentiate subpopulations of defeated mice showing depression- and anxiety-like phenotypes. The present study highlights the importance of quantifying species' typical behaviors to better understand the mechanisms of social avoidance after repeated social stress.

## Materials and methods

### Animals

Male C57BL/6J (12–20 week, 25–35 g) and retired breeder Swiss (16–25 week, 35–45 g) mice were used as intruder (experimental) and resident (aggressor), respectively. All animals were provided by the animal facility of the Brain Institute of the Federal University of Rio Grande do Norte (Natal, Brazil) and were single- (Swiss) or group-housed (C57bl/6; maximum of six mice per cage) at standard conditions (23 ± 2°C, 12 h light/dark cycle, lights on at 7 a.m.). Food and water were available *ad libitum*. All procedures were in accordance with the international guidelines and the Institutional Animal Care and Use Committee at the Federal University of Rio Grande do Norte, and all protocols were approved before the beginning of the experiments (Protocol #38/2011).

### Social defeat stress

**Figure 1:**
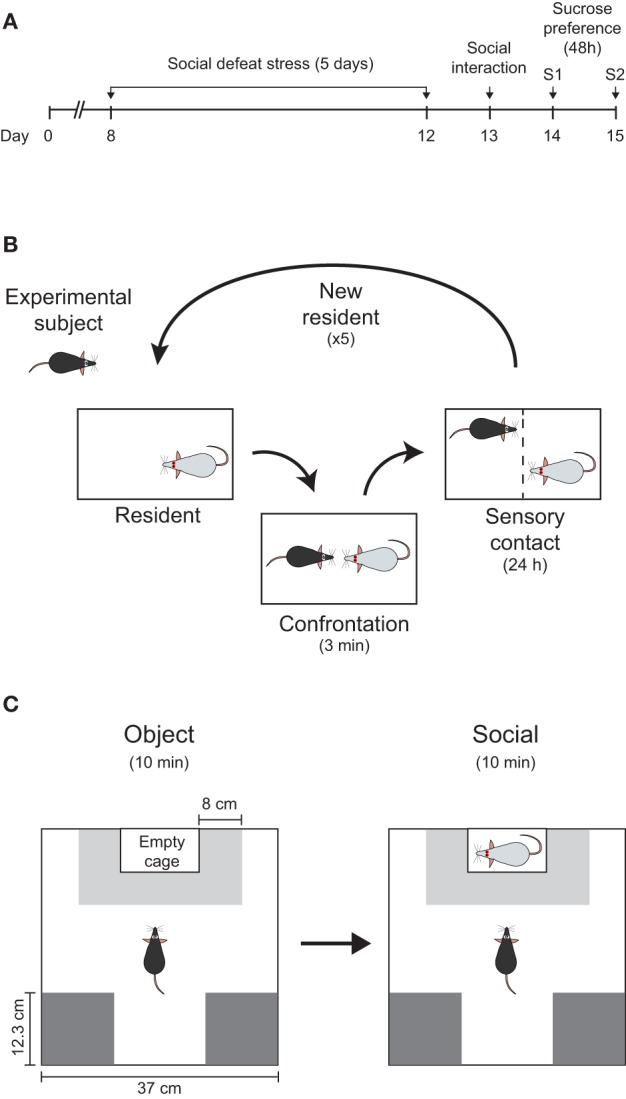
**Experimental design**. **(A)** Timeline showing all the steps of the experimental manipulations during 15 days. Animals were individually housed on day 0. **(B)** Resident–intruder paradigm was used as repeated social defeat stress (see Section Materials and Methods for a detailed description). **(C)** Scheme of the social interaction test (*object* and *social* sessions) showing the position of the restrain cage and the interaction zone (light-gray) and corners (dark-gray), as well as its respective dimensions.

We used a modified version of the resident–intruder paradigm as reported previously (Krishnan et al., 2007). The model consisted of placing an intruder (C57BL/6) mouse inside the home cage of a heavier and more aggressive resident (*Swiss*). Before the start of the experiments, *Swiss* mice were selected based on their aggressive behavior (Miczek et al., 2001). Only animals displaying attack latencies shorter than 30 s in at least two consecutive sessions (out of four screening tests) were included in the experiments. Briefly, intruder mice were exposed to a different resident mouse for no longer than 3 min each day, during five consecutive days (Figure 1A). During confrontation, resident mouse attacked the intruder within the first 30 s (average and standard deviation latency to attack: 13.5 ± 19.7 s; *n* = 81 confrontations). Preliminary experiments from our lab showed that 3 min were sufficient to allow over 30 bites from the resident and to induce sustained subordination behavior from the intruder (i.e., submissive upright, vocalization, and flight). After each confrontation, a perforated plexiglass partition was used to separate the resident's cage in two halves and the animals were in sensory contact for 24 h, until the next confrontation session (Figure 1B). Control mice experienced similar experimental condition but no physical contact occurred. Animals were handled daily and inspected for health conditions. If severe wounds were detected, animals were removed from the experiment. After the last confrontation session (5th day), animals were single-housed for the rest of the experiment.

### Social interaction test

The social interaction test used was based on the social approach-avoidance test previously described by Berton et al. (2006). All experiments took place 24 h after the last defeat during the daylight period and in a different environment of the confrontation sessions. First, animals were transferred to the new, quiet, and dimly lit room 1 h before the beginning of the test. After habituation, each animal was placed in the center of a square arena (white plexiglass open field, 37 cm each side and 30 cm high) and behavior was monitored by video (Cineplex Studio, 50 fps, camera placed above the arena). Animals were allowed to fully explore the arena twice, for 600 s in each session, under two different experimental sessions. In the first (“*object*” session), an empty perforated plexiglass cage (10 × 6.5 × 30 cm) was placed in the middle of one wall of the arena (Figure 1C). In the second session (“social” session), an unfamiliar *Swiss* male mouse was introduced into the cage as a social stimulus. Although it can be argued that the probe mouse used in the social interaction test resembles the aggressor, and this could foster social aversion, this possibility is unlikely, since previous experiments demonstrate similar amounts of social investigation irrespective to the strain (i.e., C57BL/6J; Berton et al., 2006). Before each session, the arena was cleaned with 5% alcohol solution to minimize odor cues. Between both sessions, the experimental mouse was removed from the arena, and returned to its home cage for 2 min.

### Ethological analysis of social interaction

Locomotion and arena occupancy during *object* and *social* sessions were determined using animals' horizontal position extracted by a custom-made video tracking software (MouseLabTracker; Tort et al., 2006). Conventional measures of arena occupancy, like time spent in the interaction zone and corners were quantified. The former is commonly used as social preference-avoidance index and is calculated by measuring the time spent in an 8 cm wide corridor surrounding the restrain cage. The corners were defined as two squares of similar areas in the opposite wall of the arena (Figure 1C). The Social Interaction ratio (*SIr*) was calculated as:

(1) $$\begin{array}{l} SIr=\sum TIZ_{social}/\left(\sum TIZ_{social}+\sum TIZ_{object}\right) \end{array}$$

where *TIZ*_*social*_ is the time spent in the interaction zone during the *social* session and *TIZ*_*object*_ is the time spent in the interaction zone during the *object* session. This *SIr* is a slightly modified version of the ratio used previously (Krishnan et al., 2007). Values vary from 0 to 1, where *SIr* > 0.5 indicates preference for social interaction and *SIr* < 0.5 indicates social avoidance.

Further ethological analyses were performed with the assistance of custom-made routines written in MatLab (Mathworks) and commercial software (Cineplex, Plexon Inc.). Occurrence, start and end, duration, frequency, and time course of investigative and defensive behaviors were determined. Social investigation bouts started when, within the interaction zone, animals' snouts got in contact with the surface of the perforated plexiglass cage while maintaining their heads directed to the inside of the cage and they ended when animals faced another direction. We used the stretched-attend posture evolution during social investigation to calculate an Approach Index. This index uses the investigation distance, i.e., the distance between the cage and animal's center of mass during social investigation, to infer the level of anxiety during investigation. For that, we first averaged the onset distance to the cage (*Don*_*k*_) for all *K* investigation bouts of animal *i*:

(2) $$\begin{array}{l} avgDon_{i}=\frac{1}{K}\overset{K}{\underset{k=1}{\sum }}\left(Don_{ki}\right) \end{array}$$

Then, we normalized the *avgDon*_*i*_ for all animals (*N*) from control and defeated groups:

(3) $$\begin{array}{l} NormDon=avgDon_{i}/\frac{1}{N}\sum_{i=1}^{N}\left(avgDon_{iN}\right) \end{array}$$

The Approach Index (*AI*) for each mouse *i* was calculated as:

(4) $$\begin{array}{l} AI_{i}=\frac{1}{K}\overset{K}{\underset{k=1}{\sum }}\left(Don_{ki}-Doff_{ki}\right)/NormDon \end{array}$$

where *Don*_*ki*_ and *Doff*_*ki*_ are the investigation distances at the onset and offset of the investigation bouts, respectively, for animal *i*. Approaches during social investigation bouts (i.e., reduction in the stretched-attend posture) yields high values of *AI*. Based on population distribution, we *post-hoc* defined *AI* = 0.6 as the threshold to separate subpopulations of defeated animals. Thus, *AI* < 0.6 was interpreted as sustained anxiety-like state during social investigation.

Flight behaviors were manually scored when the animal suddenly retreats from the social interaction zone and runs toward the corners, as previously described (Grant and Mackintosh, 1963; Blanchard and Blanchard, 1988; Eilam, 2005). Averaged animal's velocity in 200 ms bins was used to assist the scoring procedure. To determine whether flight behavior increases or decreases in the 10 min long session, we applied a linear fit model to flight occurrences in 150-s bins and determined the slope (coefficient of regression) of flight dynamics within a session. This value was used as a Flight Index (*FI*). A *FI* < 0 indicates that flight occurrence decreases over time, while a *FI* > 0 indicates sustained flight occurrence throughout the session (i.e., no habituation or adaptation).

The time spent in the center of the arena during social investigation was used as an indirect measure of anxiety (Belzung and Griebel, 2001) and sucrose intake (see below) was used as a measure of anhedonia (Papp et al., 1991). Both measures were used to compared defeated animals with low (< 0.6) and high (>0.6) *AI* and positive and negative value of *FI*. This analysis was used to cross-validate our indexes. All behavioral measures were averaged for four consecutive epochs of 150-s, for both *object* and *social* sessions (chunks of 30-, 50-, and 300-s duration yielded similar results and are not shown).

### Sucrose-preference test

Twenty-four hours after the social interaction test, all animals were allowed to choose between 1% sucrose solution and water for 48 h. Sucrose preference was calculated as a ratio of sucrose intake to the total amount of liquid intake. It was used as an index of the hedonic state (Papp et al., 1991) and compared between conditions and according to the behavioral indexes described above. Solutions were filled in 50 ml tubes, renewed and weighed daily, at 8 a.m. Animals were not food deprived before or during the experiment and the positions of the tubes in the cage were interchanged at each 12 h to account for drinking place preferences.

### Statistical analysis

All behavioral and statistical analyses were performed using custom-made routines in MatLab (Mathworks). Normality and variance homogeneity were verified using Kolmogorov–Smirnov's and Bartlett's tests, respectively. Levene's test was used to compare the variability of distributions (represented as the coefficient of variation) between groups. Statistical analyses of behavioral parameters were performed using one- or two-way ANOVA with repeated measures considering group (control vs. defeat) as the independent factor, and session (*object* vs. *social*) and time (0–150, 150–300, 300–450, 450–600 bins) as the within-groups factors. This approach allowed the investigation of possible interactions between factors. Non-parametric comparisons were made using Mann-Whitney U-test. Chi-square was used to compare proportions. Statistical significance was set at 5% and Bonferroni correction for multiple comparisons was applied. Data are expressed as mean ± S.E.M unless otherwise specified.

## Results

### Defeated mice exhibited initially transient period of social avoidance during extended sessions of social interaction

**Figure 2:**
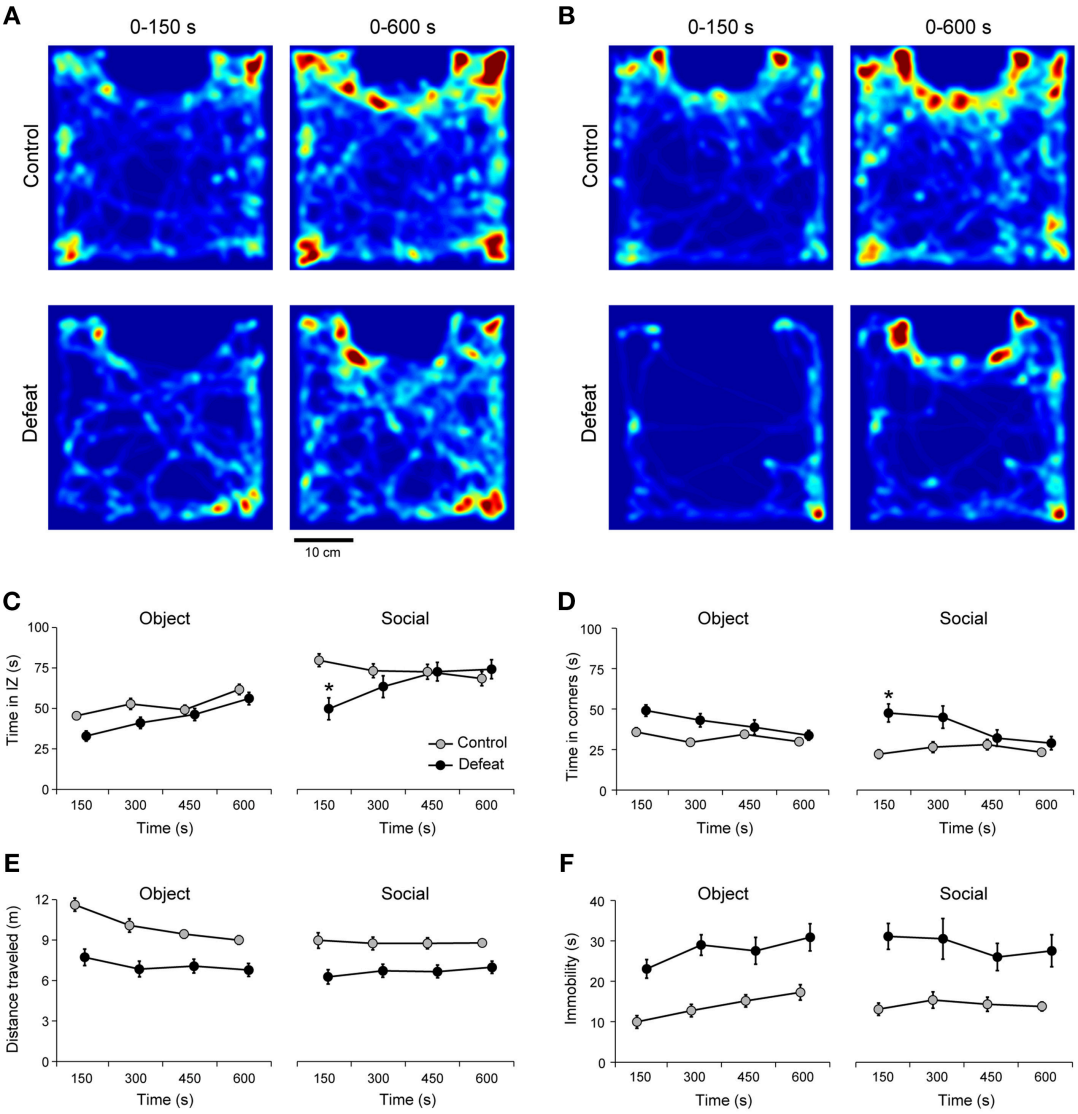
**Transient social avoidance after social defeat stress**. Heat map of arena occupancy for representative control (upper panels) and defeated (lower panels) mice at two different time points (after 150 s and 600 s) of the *object* **(A)** and *social* **(B)** sessions. Warm and cold colors represent high and low occupancy rates, respectively (same color bars for all Figures). Note that repeated social defeat stress modifies occupancy maps (lower panels) leading to avoidance of the interaction zone during *social* session, mainly in the first time bin (150-s). The time spent in the interaction zone **(C)** and corners **(D)**, as well as the locomotion **(E)** and immobility **(F)** during *object* and *social* sessions for controls (*N* = 30) and defeated (*N* = 36) mice. IZ: interaction zone. ^*^P < 0.05 control vs. defeated in the same time bin of the *social* session. Bonferroni *post-hoc* test. Data points of the control and defeated groups are slightly shifted within each time bin for clarity purposes.

It was recently argued that behavioral measures can change significantly in time (Fonio et al., 2012b). Here, time-dependent variation in social behavior induced by repeated social defeat stress was assessed in extended, 10 min long sessions, of the social interaction test. During the *object* session, defeated mice did not differ from controls in the general pattern of arena occupancy (Figure 2A). However, in the presence of an unfamiliar conspecific (*social* session), animals from the control group spent more time in the interaction zone, whereas defeated mice spent less time in the interaction zone (Figure 2A). This difference was statistically significant in the first 150-s bin for the time in the interaction zone [Figure 2B; Time vs. Group interaction: *F*_(3, 512)_ = 3.72; *P* < 0.01] and corners [Figure 2C; Time vs. Group interaction: *F*_(3, 512)_ = 3.69; *P* < 0.01]. After 150 s, the occupation of the interaction zone and corners were similar for controls and defeated mice (Figures 2B,C). The initial transient difference in arena occupation was observed only in the presence of an unfamiliar conspecific, but not during *object* session [Figure 2B; Time vs. Session interaction: *F*_(3, 512)_ = 2.07; *P* = 0.19, and Figure 2C; Time vs. Session interaction: *F*_(3, 512)_ = 0.61; *P* = 0.60].

Repeated social defeat stress induced a markedly decrease in locomotor activity [Figure 2D, main effect for Group: *F*_(1, 512)_ = 154.9; *P* < 0.001] and increased immobility [Figure 2E; main effect for Group: *F*_(1, 512)_ = 96.6, *P* < 0.001] during both *object* and *social* sessions. Importantly, this behavior showed no adaptation in time [main effect for Time: *F*_(3, 512)_ = 0.88, *P* < 0.44] during *object* and *social* sessions. Also, this change in exploratory pattern cannot be explained by movement impairment since average speed of locomotion as well as the highest speed achieved during *object* and *social* sessions were not different between controls and defeated mice (data not shown). Together, these observations suggest that social avoidance in defeated animals occurs mostly during the first 150 s of the test. Below we explore whether this result reflects, at least in a certain extent, novelty-induced anxiety.

### Delayed expression of social investigation behaviors in defeated mice

**Figure 3:**
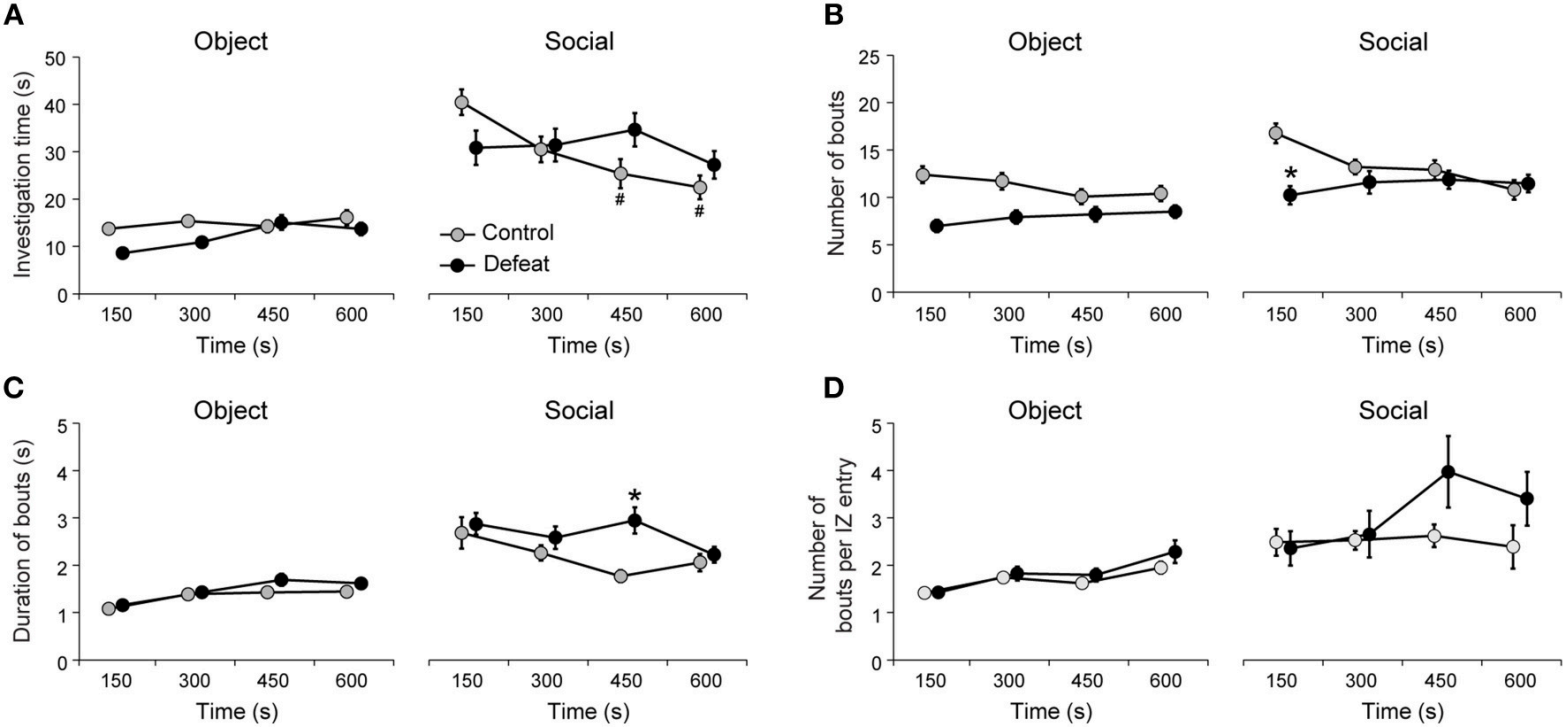
**Temporal evolution of social investigation behavior revealed delayed motivation for social interaction in defeated mice**. **(A)** Both controls and defeated mice spent more time investigating *social* than *object* stimuli, but only controls showed habituation of social investigation. **(B)** Defeated mice show initial suppression of the number of investigation bouts and delayed motivation to interact socially, as suggested by **(C)** the increased duration of social investigation bouts after 300 s. **(D)** Number of investigation bouts per interaction zone entry reveals large variation in socially motivated behavior of defeated group. ^#^*P* < 0.05 vs. in comparison to the 150-s bin of the *social* session, same group. ^*^P < 0.05 control vs. defeated in the same time bin of the *social* session. Bonferroni *post-hoc* test. Data points of the control and defeated groups are slightly shifted for clarity purposes.

To better understand how social interaction evolves in time we analyzed parameters of *object* and *social* investigation behavior, specifically the investigation time, the number of investigation bouts, the average duration of investigation bouts and the number of investigation bouts per interaction zone entry (Figure 3). Both controls and defeated mice showed increased investigation time of the *social* stimulus in comparison to the *object* stimulus [Figure 3A; main effect for Session: *F*_(1, 512)_ = 195.02, *P* < 0.001]. However, only control mice showed habituation to the presence of the unfamiliar conspecific, since the time spent investigating the social stimulus decreased after 300 s [Figure 3A, right panel; Time vs. Group interaction: *F*_(3, 512)_ = 4.70; *P* < 0.01]. Despite the general increase in time investigating the *social* stimulus in comparison to the *object* stimulus for both groups, only defeated mice showed decreased number of social investigation bouts [Figure 3B, main effect for Group: *F*_(1, 512)_ = 36.2, *P* < 0.001]. This was particularly true for the initial 150-s bin of the *social* session [Figure 3B; Time vs. Group interaction: *F*_(3, 512)_ = 7.01; *P* < 0.001]. Interestingly, defeated mice showed increased duration (average) of social investigation bouts later in the test in comparison to controls [300-s bin; Figure 3C, right panel; Time vs. Group interaction: *F*_(3, 512)_ = 2.7, *P* < 0.05]. Further, longer sessions of social interaction revealed increased variance for the number of bouts per interaction zone entry in defeated group, particularly in the 300-s and 450-s bins (Figure 3D; coefficients of variation: 42 and 49% for controls and 108 and 112% for defeated group in the 150–300 and 300–450 s time bins, respectively,*P* < 0.001 Levene's test). These results suggest that longer (10 min) sessions of social interaction can reveal subtle differences between control and defeated animals than the standard parameter of occupancy time of the interaction zone or corners. Next, we evaluated whether the expression of defensive behaviors are also modified by repeated social defeat stress.

### Risk assessment behavior during social investigation

**Figure 4:**
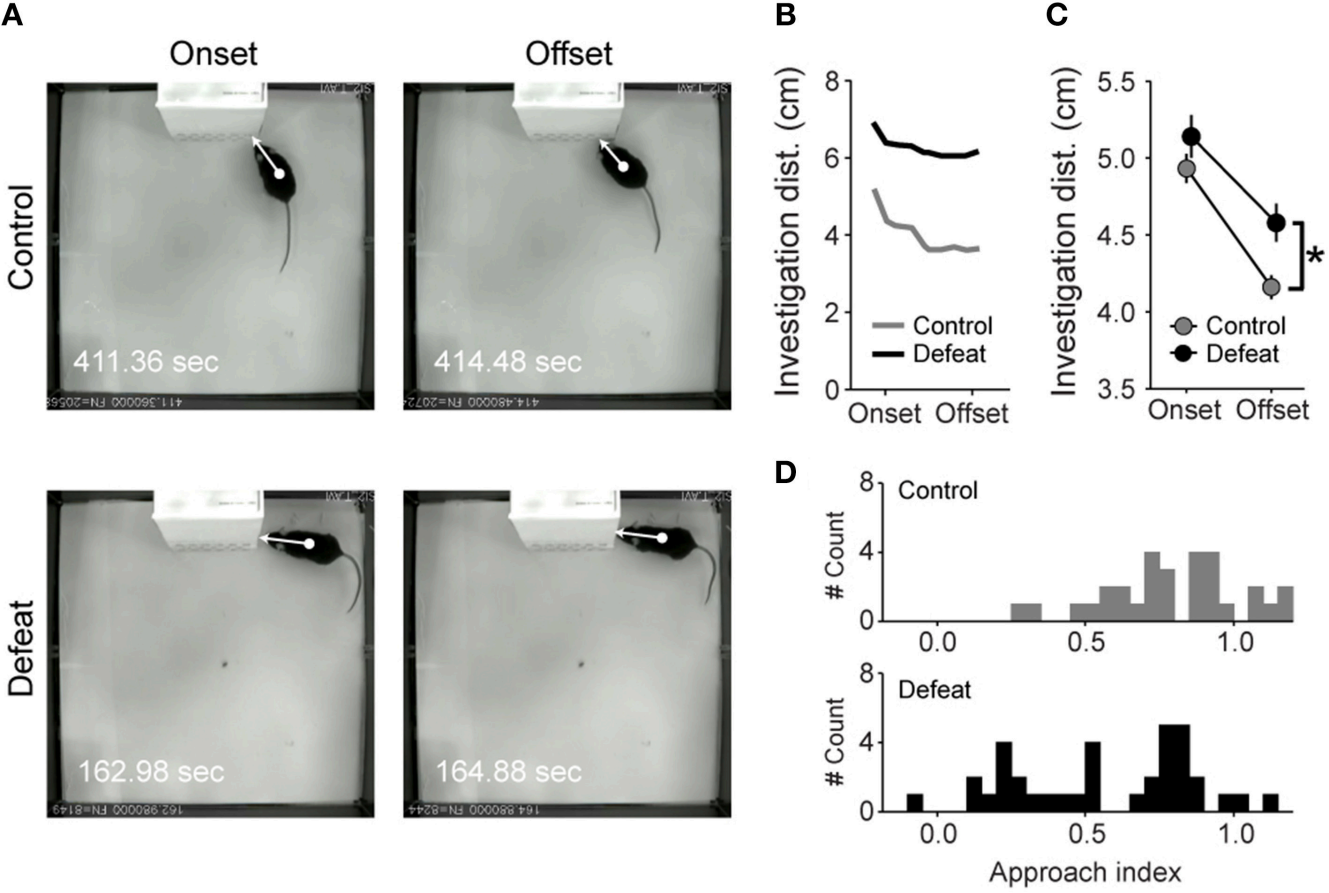
**Repeated social defeat stress leads to sustained stretched-attend posture during social investigation bouts**. **(A)** Illustrative examples of risk assessment at the onset (left) and offset (right) of social investigation in control (upper panel) and defeated (lower panel) animals. White arrows represent the investigation distance, computed as the shortest distance between animal's center of mass (circle) and the edges of the restrain cage (tip of the arrow). **(B)** Investigation distance from the onset to the offset of the social investigation bout shown in A (control and defeated animal represented by gray and black lines, respectively). **(C)** Grand averaged investigation distance at the onset and offset of social investigation bouts. Note that the distances were similar for both groups at the onset of the social investigation but defeated mice showed increased distance to cage at the offset. **(D)** Frequency distribution histograms of the Approach Index (*AI*) for control (top, gray) and defeated (bottom, black) mice. ^*^P < 0.01, Student's *t*-test.

By measuring the distance of the animals' center of mass to the borders of the restrain cage (i.e., investigation distance) during the onset until the offset of social investigation bouts, we were able to infer the degree by which the animals approach the social stimulus from the start to the end of the investigation bouts (Figure 4). Control mice progressively reduced investigation distance from the onset to the offset of social investigation bouts (upper panels in Figure 4A and Figures 4B,C). In contrast, the approach during investigation was significantly smaller in defeated mice, especially in the offset of social investigation bouts (Figure 4C). We then analyzed the extent of approach-avoidance tendencies while mice were engaged in social investigation. For this, we created an *AI* for social investigation (see Section Materials and Methods), where the value tends to be higher when the mouse initiates the investigation bout from a short distance and when it approaches the social stimulus from the onset to the offset of the social investigation bouts. If the animal initiates the social investigation from a larger distance (i.e., when it investigates the social stimulus in stretched-attend posture), or if it does not approach the restrain cage borders during the investigation bout, the *AI* tends to be lower. As expected, control mice consistently approached the social stimulus during social investigation, an effect evidenced by the high *AI* values (upper histogram in Figure 4D; *AI* = 0.79 ± 0.04), while defeated mice did not (*AI* = 0.56 ± 0.06, *P* < 0.01; Mann-Whitney U-test). Furthermore, defeated mice showed higher inter-individual variability in the *AI* (coefficients of variation: 29.2 and 53.3% for controls and defeated groups, respectively; *P* < 0.05; Levene's test). For further analysis, a threshold of *AI* = 0.6 was set according to its distribution and this value was used to separate defeated animals with low and high levels of anxiety during social investigation (see below).

### Flight behavior after social investigation

**Figure 5:**
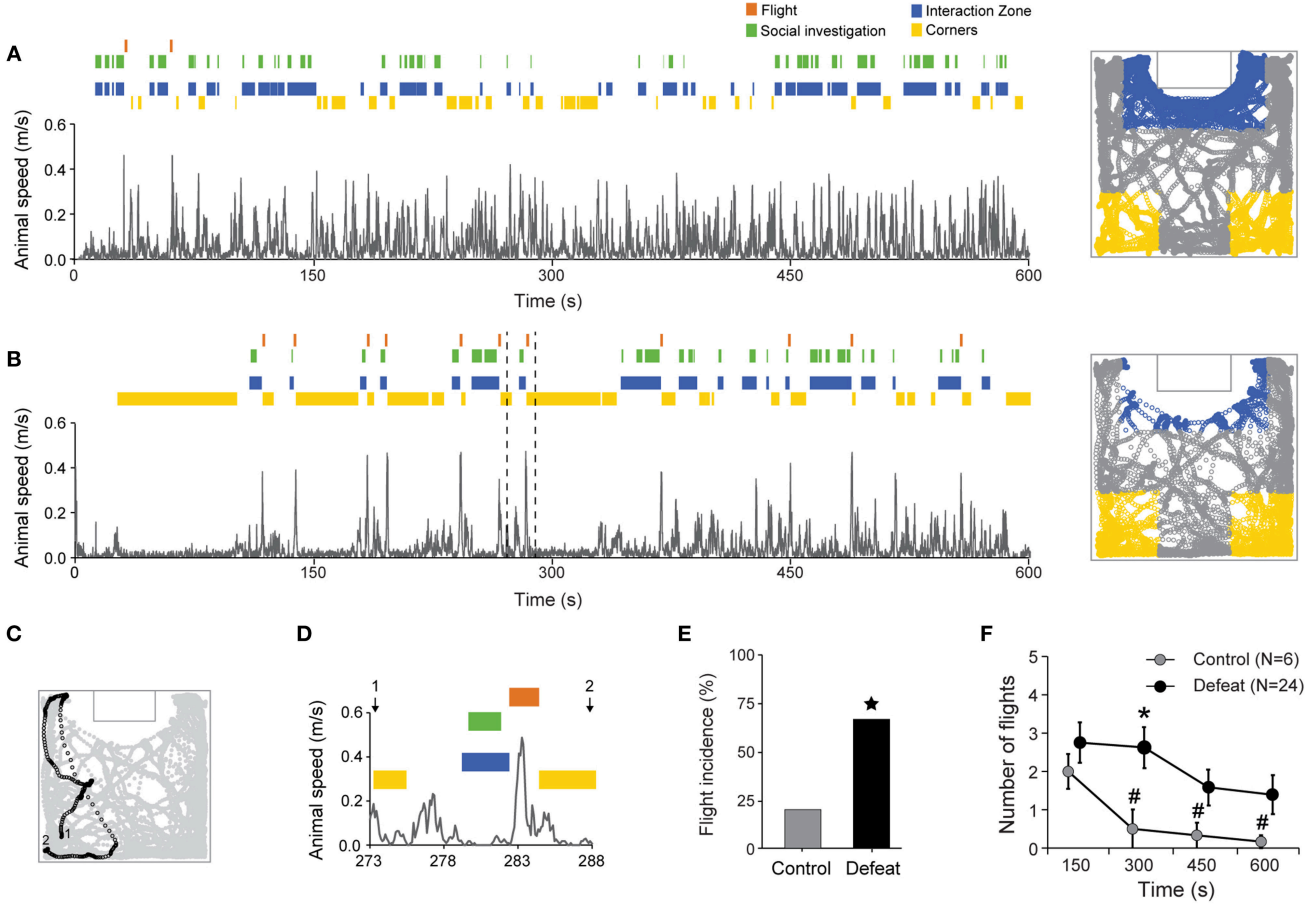
**Increased flight behavior occurrence after social defeat stress**. Temporal evolution of speed superimposed with the raster plot of behavioral events in one representative control **(A)** and defeated **(B)** animals. Flight behavior was scored when an abrupt increase in animal's velocity take place when moving from the interaction zone to the corners. Right panels depict video tracking data for control **(A)** and defeated **(B)** animals during the *social* condition. The tracks within the corners and interaction zone areas are shown in yellow and blue, respectively. **(C,D)** Animal's positions and the respective time-course of a typical approach followed by flight behavior (dashed line in **B**). **(E)** Flight incidence in control and defeated animals. **(F)** Number of flights over time during the *social* session. Only animals with at least one flight were considered (Controls: *N* = 6; Defeated: *N* = 24). ^
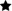
^*P* < 0.001, Chi-square test. ^*^P < 0.01, control vs. defeated in the same time bin; ^#^*P* < 0.01, in comparison to the 150-s bin, same group. Data points of the control and defeated groups are slightly shifted for clarity purposes.

Flight behavior was characterized by abrupt and high speed ambulation away from the interaction zone toward one of the corners. Animals' speed and selected behavior timestamps over time during *social* session are shown in Figures 5A,B, for representative control and defeated mice, respectively. Bursts of fast locomotion after brief forays into the interaction zone (blue tags) and social investigation bouts (green tags) followed by corners occupation (yellow tags) can be clearly identified in the video-tracking data (right panels in Figures 5A,B). An illustrative flight behavior is shown in Figures 5C,D (dashed lines in Figure 5B). In this example, the animal started (1) by moving from the left corner toward the interaction zone where it engaged in social investigation. After the end of the social investigation bout, the animal abruptly retreated and fled back toward the corner (2) (Figures 5C,D). Although the maximum speed during flights did not differ between controls and defeated mice (mean ± SEM: 34.0 ± 1.5 and 34.5 ± 0.7 cm/s, respectively; *P* = 0.84, Student's *t*-test), the incidence of flights was higher in the defeated group (67% [24 out of 36]) in comparison to controls (20%, [6 out of 30]; *P* < 0.001, Chi-Square Test; Figure 5E). Also, the total number of flights (considering only those animals which presented it) was higher in defeated animals in comparison to controls [main effect for Group: *F*_(1, 256)_ = 39.32, *P* < 0.001]. Additionally, flight occurrence decreased in time [main effect for Time: *F*_(3, 256)_ = 5.48, *P* < 0.01] but it dropped faster in control than defeated animals [Time vs. Group interaction: *F*_(3, 256)_ = 2.89, *P* < 0.05; Figure 5F]. As observed for social investigation, the temporal dynamics of flight behavior occurrence was also highly variable within the defeated group, and this observation is further explored below.

### Defeated mice showed distinct patterns of flight occurrence over time

**Figure 6:**
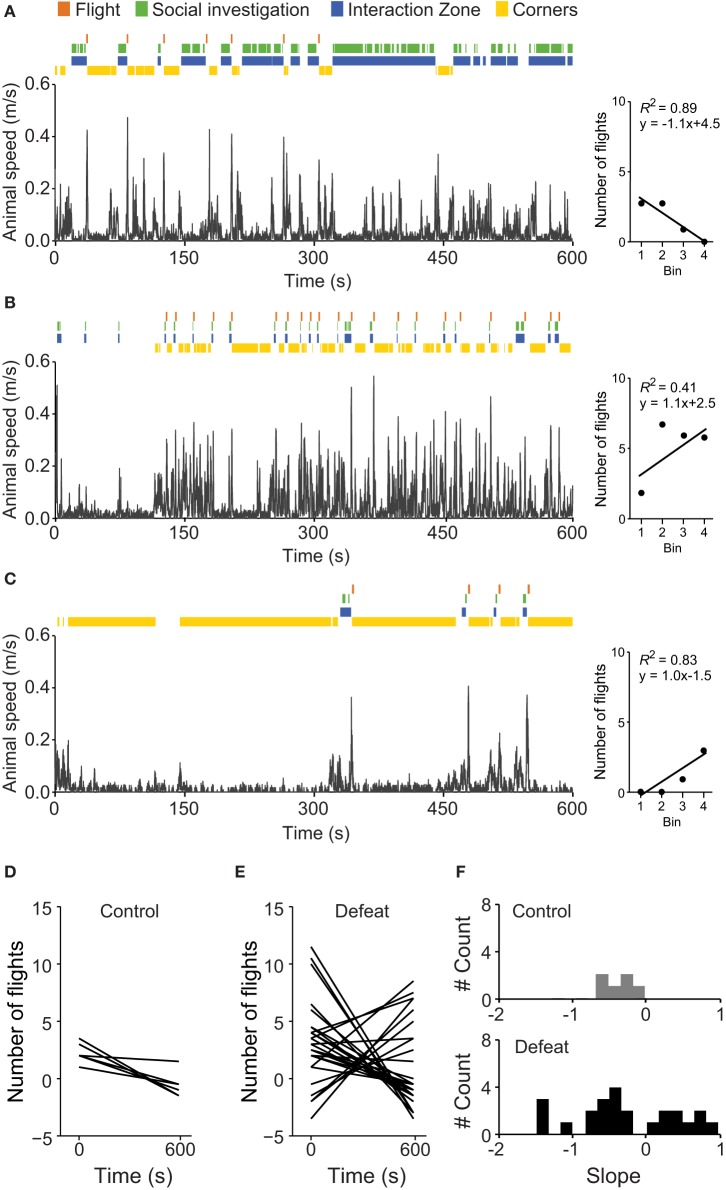
**Defeated mice display distinct patterns of flight occurrence over time**. Illustrative examples of the temporal evolution of exploratory behavior during the social interaction test (*social* session) for defeated animals showing attenuated **(A)**, sustained **(B)**, and delayed **(C)** flight occurrence. Fitted curves for the number of flight occurrences in the respective examples are shown in the right graphs (x-axis are in bins and insets show the *R*-square and fitted equations). Control animals (Figure 5A) show decreased flight occurrence after 150-s, while flight behavior decreases more slowly in socially defeated mice. Fitted curves for the number of flight occurrences in control **(D)** and defeated **(E)** mice revealed strong variation in flight behavior in defeated, but not control, mice. **(F)** Frequency distribution histograms of the fitted slope (Flight Index) of control (top) and defeated (bottom) mice.

By visually inspecting the time-course of social investigation and flights, as well as arena occupation, we noticed considerable inter-individual variation in coping strategies among defeated animals. Although some individuals of this group initially showed active avoidance responses (social investigation bouts followed by flights), in the second half of the *social* session, they switched to a strategy of uninterrupted social interaction (Figure 6A). Differently, some individuals displayed a recurrent pattern of social investigation followed by flight, associated with increased time spent in corners (Figure 6B). We also observed individuals with initially long lasting passive avoidance that switched to active responses only in the second half of the *social* session (Figure 6C). To determine if the number of flights was decreasing or increasing during the session, we linearly fit the total number of flights in the four consecutive 150-s bins and calculated the slope of the fitted curve (right graphs in Figures 6A–C). If the number of flights decreased during the 600 s session, we expected a negative slope. Figures 6D,E depict the linear fitted curves for those animals with two or more flights in the control (*N* = 6) and defeated (*N* = 24) groups, respectively. Figure 6F shows the frequency distribution histogram for the fitted slopes. All control mice decreased flight behavior during the *social* session. However, a considerable heterogeneous pattern was observed for defeated animals, suggesting the existence of at least two subpopulations of defeated mice regarding active strategies to cope with potential stressful situations. For the following analysis, we considered the calculated slope of flight occurrence as the *FI* and a value of 0 (zero) was established as the threshold to separate defeated animals in two subpopulations.

### Segregation of defeated mice into susceptible and resilient subpopulations

**Figure 7:**
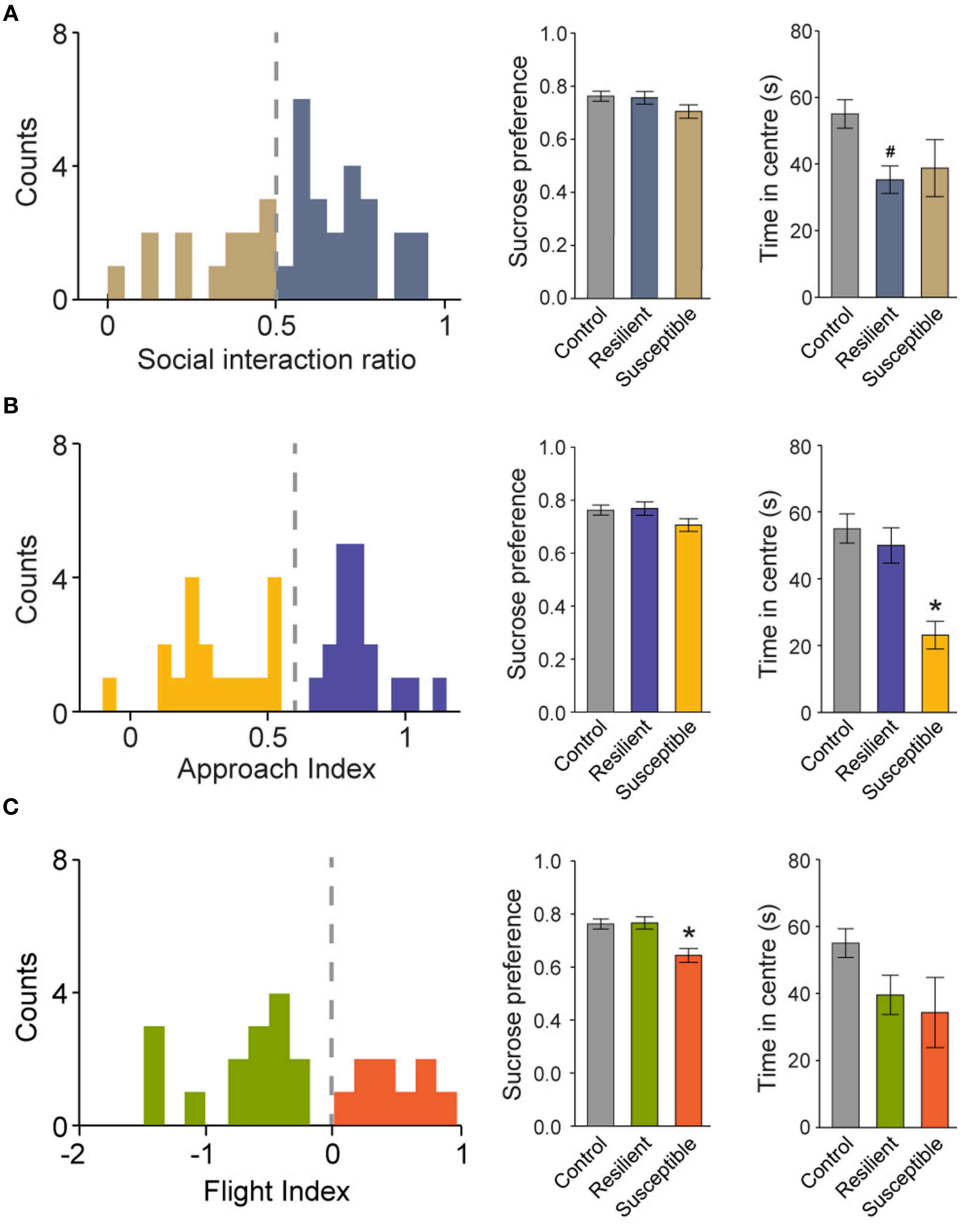
**Segregation of defeated mice into susceptible and resilient subpopulations using different indexes**. Left panels show the frequency distribution histograms for the *SIr* **(A)**, *AI* **(B)** and *FI* **(C)**. Middle and right panels show the sucrose preference and the total time spent in the center of the arena during *social* session, respectively. For a detailed criteria used to determine resilient and susceptible mice, please refer to the text. ^#^*P* < 0.05 vs. control only. ^*^P < 0.05 vs. both control and resilient groups. Bonferroni *post-hoc* test.

Traditionally, socially defeated mice are classified as resilient and susceptible using the *SIr*, or some variation of the index, in 150-s long sessions (Berton et al., 2006; Golden et al., 2011). This labeling can be further verified using the sucrose preference test. Here, defeated mice drank 20% less sucrose than controls (absolute sucrose intake in 48h, in grams: 15.7 ± 0.8 and 12.5 ± 0.7, for control and defeated groups, respectively; *P* < 0.05, Student *t*-test). Importantly, no difference in water intake was observed (in grams, 4.8 ± 0.3 and 4.8 ± 0.3, for control and defeated groups, respectively; *P* = 0.86, Student *t*-test). We also computed the *SIr* for the first 150-s of the sessions to show that 33% (*N* = 12/36) of defeated mice displayed *SIr* < 0.5 and therefore, could be classified as susceptible (Figure 7A; left panel). Using this classification, we compared the sucrose preference and the time spent in the center of the arena in susceptible and resilient animals. Surprisingly, both subpopulations did not differ in the sucrose preference test (Figure 7A; middle and right panels). Conversely, only the *resilient* subgroup of defeated animals spent less time in the center of the arena during *social* session (Figure 7A; right panel). This observation suggests that behavioral phenotyping based exclusively on interaction zone occupancy during 150-s long sessions can be prone to misclassification.

We then tested whether the previous presented indexes, namely the *AI* and the *FI*, could better separate subpopulations of resilient and susceptible mice of the defeated group. Animals with an *AI* below 0.6 spent significantly less time in the center zone of the arena (Figure 7B; right), but no differences in sucrose preference were observed (Figure 7B; middle). This measure allowed the identification of a subpopulation of susceptible mice (50%; 18 out of 36) only for anxiety-related behaviors. On the contrary, the *FI* permitted the identification of a subgroup of anhedonic animals (25%; 9 out of 36) in defeated mice (Figure 7C; middle). These animals did not differ in the amount of time spent in center of the arena (Figure 7C; Right), suggesting that the time-dependent expression of flight behavior during social interaction can be used as a reliable measure to classify depressive-like behavioral traits. Finally, using both indexes, we observed that 14% (5 out of 36) of the defeated group expressed both depressive- and anxiety-related symptoms.

## Discussion

Previous studies have reported significant changes in social interaction after social defeat stress (Kudryavtseva et al., 1991; Krishnan et al., 2007; Razzoli et al., 2011; Venzala et al., 2012; Challis et al., 2013; Friedman et al., 2014). Here, we corroborate and extend these findings by showing that repeated (5 days) defeat interspersed by continuous sensory contact with the resident-aggressor led to social avoidance. Surprisingly, the pattern of social investigation changed during our 10 min long session; while control animals reduced investigative behavior along the session, defeated mice increased it. Also, variability in behavior investigation patterns was much higher in the defeated group in comparison to controls. Quantifying the temporal evolution of risk-assessment and flight behaviors during social investigation allowed us to grasp part of the myriad of exploratory repertoire after social stress. Using these ethological tools, we present two new indexes that can be of use to separate anxiety- and depressive-related phenotypes after social stress. The indexes can have important consequences in the interpretation of biological data regarding resilience and susceptibility to stress. Below, we discuss the implications of these ideas for the search of a biological basis of human stress-related disorders.

### Social avoidance behavior after social defeat

Chronic exposure to stress can lead to long-lasting changes in behavior (McEwen, 2012). Particularly, social defeat stress, which combines varied levels of both physical and psychological stress, can result in anhedonia and social avoidance, as well as metabolic disturbances in humans (Hawker and Boulton, 2000; Björkqvist, 2001) and rodents (Kudryavtseva et al., 1991; Berton et al., 2006; Krishnan et al., 2007; Bondar et al., 2009; Walsh et al., 2014). This has prompted the general idea that chronic social defeat stress in mice is a suitable animal model to study stress-related psychiatric disorders, such as anxiety and depression (Bartolomucci et al., 2009; Nestler and Hyman, 2010; Hollis and Kabbaj, 2014; Czéh et al., 2016). In this respect, it has been extensively shown that social withdrawn and decreased sucrose intake in socially defeated animals can be reversed by chronic treatment with antidepressants (Berton et al., 2006; Rygula et al., 2006; Krishnan et al., 2007; Venzala et al., 2012). However, it has been suggested that anxiety can modify the behavioral outcome of both social interaction (Allsop et al., 2014) and sucrose preference tests (Bondar et al., 2009), calling for new approaches toward social behavior quantification (Peters et al., 2015).

Social avoidance is a natural, adaptive and complex behavior which allows the individual to deliberately withdraw from (potential) unpleasant situations (Blanchard et al., 2005). Exaggerated and sustained avoidance has long been considered a pathological symptom (Charney and Manji, 2004; Southwick et al., 2005). It may arise from the disrupted motivational processes related to social interaction or from the activation of the neuronal pathways associated with fear identification and responses toward social stimulus (Steimer, 2011; Toth et al., 2012; Toth and Neumann, 2013). Thus, social avoidance reflects both a “depressive state” and an “anxiety state.” Sucrose intake has also been prone to bias, since gustatory and olfactory transduction, familiarity, context of liquid intake and group comparisons can all modify the preference index (Bondar et al., 2009).

Despite these concerns, important contributions to the dopaminergic theory of stress-related disorders have been put forwarded by the combination of social defeat stress model and social interaction test (Berton et al., 2006; Krishnan et al., 2007; Walsh et al., 2014), but not without contradictory results (Tye et al., 2013). In the following sessions, we argue that careful quantification of defensive behaviors in long observational sessions can be of certain value in the resolution of such contradictions.

### Disentangle anxiety- and depression-related behaviors during social interaction

One obvious question when one considered disentangling anxiety- and depression-related behaviors is whether they represent two distinct clinical entities or different symptoms of a single, broad-spectrum, psychiatric illness. In this respect, comorbidity, the complex overlap between manifestations of different disorders, has long been recognized in individuals suffering from both anxiety and depression (Gorman, 1996; Mineka et al., 1998). Also, prolonged stress can trigger both (Gorman, 1996; Mineka et al., 1998; Waugh et al., 2012). Therefore, comorbidity introduces substantial difficulties in the diagnosis, treatment, and understanding of the biological substrates of such pathologies. The ethological analysis presented here suggests that intermingled anxiety- and depression-related behaviors can be separated in mice exposed to social defeat by looking at defensive behaviors during social interaction test (see Figure 7). This approach identified 50, 25, and 14% of the defeated animals with anxiety-, depression-related, or both phenotypes, respectively, indicating that the model of repeated social defeat stress used here is more likely to produce anxiety. Also, this experimental approach can be used to model the clinical concept of “anxious depression,” a separate diagnostic class that combines symptoms of anxiety disorders and major depression (Lydiard and Brawman-Mintzer, 1998). Further studies using antidepressants and anxiolytics will be necessary to demonstrate the predictive validity of these indexes and to better characterize the behavioral changes associated with social stress.

### Learning to be fearless

Several lines of evidence suggest that during stretched-attend posture the animal gathers information about impending stimulus (Blanchard et al., 2011). When the possible menaces are explored or becomes familiar to the animal, they switch to non-defensive and adaptive behaviors, like foraging and free exploration. Therefore, stretched-attend posture can be interpreted as reflecting animal's state of apprehension which, at least from the phenomenological point of view, would relate to human anxiety (Blanchard et al., 2001b). In fact, stretched-attend postures are decreased after administration of anxiolytics, such as diazepam and buspirone (Blanchard et al., 2001a; Bilkei-Gorzo et al., 2002), but not after traditional antidepressants (Kaesermann, 1986; Molewijk et al., 1995; Varty et al., 2002). We hypothesize that, during social investigation bouts, mice would express stretched-attend posture as long as the social stimulus is perceived as a potential threat. Here, we showed that defeated group displays two roughly defined distributions of the *AI* (see Figure 7B) while expressing a sustained pattern of stretched-attend posture during social investigation bouts (see Figure 4). Indeed, susceptible animal (*AI* < 0.6; Figure 7B) spent less time in the center of the arena, a measure commonly associated with anxiety (Belzung and Griebel, 2001; Fonio et al., 2012b). Notably, no difference in sucrose consumption was observed between resilient and susceptible mice separated by this index. Together, this result suggests that *AI* is a potential score to reliably segregate a subpopulation showing predominantly anxiety-like phenotype in the social interaction test.

We also identified another defensive behavior during social interaction. Flight behavior was scored when abrupt retreats and fast running soon after social investigation bouts were observed (Blanchard and Blanchard, 1988; Blanchard et al., 1999). Under our experimental conditions, this natural behavior (Calatayud et al., 2004; Yilmaz and Meister, 2013) was weakly triggered in controls (see Figure 5A). However, social defeat stress increased its incidence and delayed its habituation (see Figure 5F). Again, we have noted a large inter-individual difference in the pattern of flight occurrence (see Figure 6). Some individuals displayed risk assessment and flight behaviors in the first tens of seconds of the *social* session and soon ceased to flee, while others exhibited repeated behavioral sequences of arrest followed by flight throughout the *social* session. Since behavioral flexibility in coping with a stressor is a feature of resilience (Feder et al., 2009), we assert that *FI* is a valuable index to identify susceptible animals to depressive-like state after social stress, as suggested by the sucrose preference test (Figure 7C, but also Koolhaas et al., 1999, 2006). This idea resonates with earlier reports showing that mice exposed to context previously associated with danger (e.g., predators, aggressive conspecifics) initially freeze, then display risk assessment that gradually vanishes away giving rise to non-defensive behaviors (Blanchard and Blanchard, 1988).

### Short sessions restrict temporal unfolding of social behaviors

Increasing evidence demonstrate that short duration behavioral assays can be prone to error (Fonio et al., 2012a,b; Hager et al., 2014). In one study, the phenotypic identification of anxiety-related behavior in two different strains of mice using the open-field was dependent on the duration of the test (Fonio et al., 2012a). We have hypothesized that novelty-induced anxiety can be an important factor determining social avoidance in shorter sessions of social interaction. Long test durations can increase the opportunity to characterize individual differences in coping strategies adopted by the experimental animals to overcome behavioral challenges (Hager et al., 2014). By increasing the social interaction test sessions four-fold, we demonstrated that social defeated mice showed a brief but transient period of social avoidance at the beginning of the test (Figures 2, 3). Notably, in most defeated animals, this behavioral pattern was reversed after 150 s, which turns out to be the maximal observational period used in many studies of social interaction (Krishnan et al., 2007; Venzala et al., 2012; Chaudhury et al., 2013; Friedman et al., 2014; Iñiguez et al., 2014). This observation favors the interpretation that social avoidance in shorter sessions is more likely to reflect anxiety- than depression-related symptoms. In fact, like controls, our group of defeated mice clearly prefer *social* over *object* stimuli (Figure 3A). Interestingly, investigation decreases over the 600-s long session only for the control group, suggesting that habituation took place (Figure 3A). From the presented experiments, we could not determine whether defeated animals had some sort of rebound effect produced by novelty-induced avoidance or whether the lack of habituation relates to other cognitive alterations triggered by social defeat stress.

We also observed increased variance in the number of social investigation bouts per interaction zone entry in defeated animals (see Figure 3D). This effect was only observed at the second half (5 min) of the social interaction test, and it suggests that animals differently habituate / recover from novelty-induced avoidance. These results suggest that the heterogeneity in motivational processes associated to social behaviors can only be observed in extended sessions of social interaction.

## Conclusions

In conclusion, our results suggest that phenotypic classification based exclusively on the amount of time spent in the interaction zone in short duration sessions may be inappropriate, mainly when evaluating depression-like behaviors in socially defeated animals. Behavioral measurements of social avoidance are influenced by novelty-induced anxiety and therefore can bias quantification of the drive to social interaction. We propose that ethological approach combined with longer sessions can overcome this limitation. Here, we have introduced two new variables, namely the *Approach Index* (*AI*) and the *Flight Index* (*FI*), that are easy to quantify and informative about the anxiety- and depression-like profile after repeated social defeat stress. Further studies using pharmacological treatment are necessary to completely demonstrate the full application of the presented indexes for the screening of drugs for mood disorders.

## Author contributions

AH performed the experiments. AH and CQ conceived, designed, analyzed, and wrote the manuscript.

## Funding

This work was funded by CNPq (480875/2012-0) and FAPERN. AH was a recipient of a graduate fellowship from CAPES.

### Conflict of interest statement

The authors declare that the research was conducted in the absence of any commercial or financial relationships that could be construed as a potential conflict of interest.
